# HDAC inhibitors suppress protein poly(ADP-ribosyl)ation and DNA repair protein levels and phosphorylation status in hematologic cancer cells: implications for their use in combination with PARP inhibitors and chemotherapeutic drugs

**DOI:** 10.18632/oncotarget.28278

**Published:** 2022-10-14

**Authors:** Benigno C. Valdez, Yago Nieto, Bin Yuan, David Murray, Borje S. Andersson

**Affiliations:** ^1^Department of Stem Cell Transplantation and Cellular Therapy, University of Texas MD Anderson Cancer Center, Houston, TX 77030, USA; ^2^Department of Experimental Oncology, Cross Cancer Institute, University of Alberta, Edmonton, AB T6G 1Z2, Canada

**Keywords:** poly(ADP-ribosyl)ation, HDAC inhibitors, PARP inhibitors, chemotherapy, hematologic malignancy

## Abstract

The therapeutic efficacy of histone deacetylase inhibitors (HDACi) for hematologic malignancies and solid tumors is attributed to their ability to remodel chromatin, normalize dysregulated gene expression, and inhibit repair of damaged DNA. Studies on the interactions of HDACi with PARP inhibitors in hematologic cancers are limited, especially when combined with chemotherapeutic agents. Exposure of hematologic cancer cell lines and patient-derived cell samples to various HDACi resulted in a significant caspase-independent inhibition of protein PARylation, mainly catalyzed by PARP1. HDACi affected the expression of PARP1 at the transcription and/or post-translation levels in a cell line-dependent manner. HDACi-mediated inhibition of PARylation correlated with decreased levels and phosphorylation of major proteins involved in DNA repair. Combination of HDAC and PARP1 inhibitors provided synergistic cytotoxicity, which was further enhanced when combined with a chemotherapeutic regimen containing gemcitabine, busulfan and melphalan as observed in lymphoma cell lines. Our results indicate that the anti-tumor efficacy of HDACi is partly due to down-regulation of PARylation, which negatively affects the status of DNA repair proteins. This repair inhibition, combined with the high levels of oxidative and DNA replication stress characteristic of cancer cells, could have conferred these hematologic cancer cells not only with a high sensitivity to HDACi but also with a heightened dependence on PARP and therefore with extreme sensitivity to combined HDACi/PARPi treatment and, by extension, to their combination with conventional DNA-damaging chemotherapeutic agents. The observed synergism of these drugs could have a major significance in improving treatment of these cancers.

## INTRODUCTION

Histone acetylation is an epigenetic modification, catalyzed by histone acetyltransferases, where positively charged lysine residues at the N-terminal tails of histones are acetylated, consequently decreasing their interactions with the negatively charged DNA and relaxing the chromatin structure. Relaxed chromatin is generally associated with increased transcriptional activation [1]. This process is reversed by histone deacetylases (HDACs) which catalyze the removal of the acetyl group resulting in a transcriptionally deactivated condensed chromatin. The dynamic process of histone acetylation/deacetylation may also cause structural changes in distant locations in the chromosome and contribute to a more global effect on gene expression and other cellular processes including DNA replication and cell division [2].

Overexpression of HDACs has been associated with tumorigenesis by down-regulation of tumor suppressor genes [3, 4]; hence, HDAC inhibitors (HDACi) including vorinostat (SAHA), romidepsin (Rom), panobinostat (Pano) and belinostat have been approved by the United States Food and Drug Administration for the treatment of hematologic and other malignancies [5]. These inhibitors restore appropriate gene expression, resulting in induction of cell differentiation, cell cycle arrest and apoptosis [6]. Despite their preclinical efficacy, HDACi do not seem to be clinically highly effective as monotherapy, and potentially more effective anti-tumor activity is observed when they are combined with other anti-cancer drugs [7–9]. In this context, the differential effects of HDACi on the expression of cellular drug transporters must be considered before applying them in combination chemotherapy; e.g., they are known to decrease the level of MRP1 protein and increase MDR1 in human hematologic cancer cell lines [10]. Such mechanisms may explain the lack of clinical efficacy when Rom is combined with MDR1 ligands such as doxorubicin or vincristine [11].

The efficacy of HDACi in combination chemotherapy may also be attributed to their ability to induce DNA double-strand breaks (DSBs); in fact, HDACi-mediated changes in chromatin structure directly activate the DNA damage response [12, 13]. HDACi affect the acetylation status of proteins involved in different DNA repair mechanisms and may have an impact on the genomic instability of cancer cells [14]. Despite the numerous studies on the effects of HDACi on genomic integrity, and their interactions with poly(ADP ribose) polymerase (PARP) inhibitors (PARPi) in solid tumors [15–19], analysis of their direct effects on protein poly(ADP-ribosyl)ation (PARylation), which is critical for DNA repair, warrants a more thorough study in hematologic cancers. PARylation is catalyzed by PARP enzymes which bind to DNA breaks, self-ribosylate, and recruit and PARylate DNA repair proteins [20].

In this study, we show that HDACi inhibit protein PARylation and exhibit synergistic cytotoxicity with PARPi and DNA damaging agents in various hematologic cancer cell lines and patient-derived cell samples. The results provide another level of mechanistic insight into the previously reported observations on the HDACi-mediated inhibition of DNA repair in hematologic cancers and its exploitation for therapeutic purposes.

## RESULTS

### HDACi inhibit protein PARylation in various hematologic cancer cell lines

**Figure 1:**
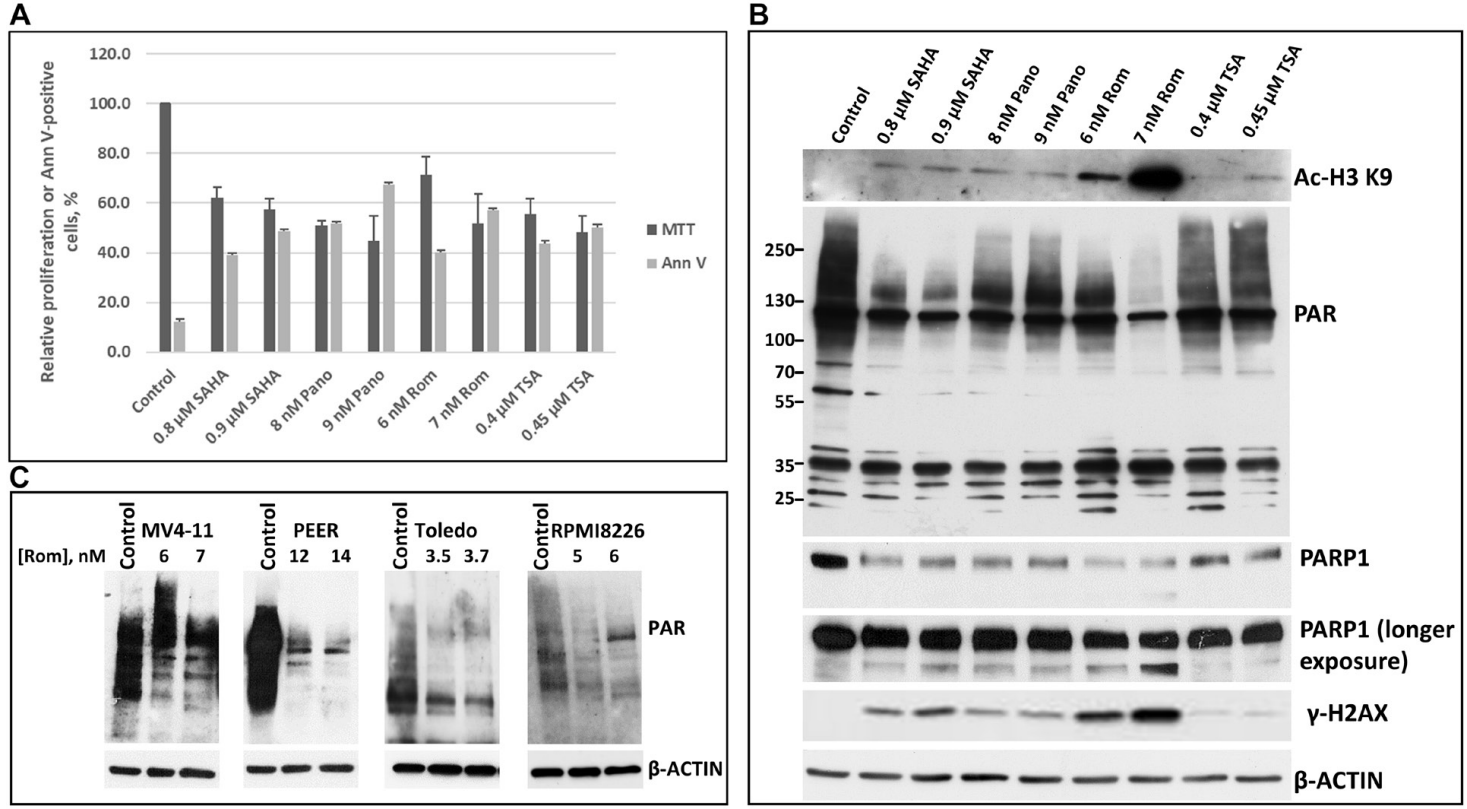
Cytotoxicity of various HDAC inhibitors and their effects on protein poly(ADP-ribosyl)ation and other modifications. MV4-11 AML cell line was exposed to two different concentrations of the HDAC inhibitors SAHA (Vorinostat), panobinostat (Pano), romidepsin (Rom) and trichostatin A (TSA) for 48 h and analyzed for cell proliferation and activation of apoptosis by MTT and Annexin V (AnnV) assays, respectively (**A**), and for protein modifications (**B**). Different cell lines were exposed to romidepsin (Rom, close to IC50) for 48 h and analyzed for poly(ADP-ribosyl)ation by Western blotting (**C**). The PAR antibody recognized PARylated proteins with 2 to 50 poly(ADP)ribose units.

We initially determined the ~IC_50_ of SAHA/vorinostat, Pano, Rom and trichostatin A (TSA) in the MV4-11 acute myeloid leukemia (AML) cell line based on cell proliferation (MTT) and apoptosis (Annexin V) assays (Figure 1A). It was apparent that all four HDACi inhibited cell proliferation and induced apoptosis over a wide range of drug concentrations (Figure 1A). We then determined the effect of these inhibitors at ~IC_50_ concentrations on histone acetylation and on the status of total protein PARylation in MV4-11 cells (Figure 1B). Of the four HDACi used in this study, Rom (at nM range) was the most efficacious in inhibiting both deacetylation of histone 3 at residue lysine 9 and protein PARylation, followed by SAHA (Figure 1B). All four HDACi down-regulated the level of PARP1 and caused slight cleavage of the enzyme, although a significant level of full-length PARP1 was still present; the DNA damage response was also activated as indicated by increased phosphorylation of H2AX (Figure 1B). The HDACi-mediated inhibition of PARylation by Rom was also observed in PEER (T-cell acute lymphoblastic leukemia: T-ALL), Toledo (diffuse large B-cell lymphoma), and RPMI8226 (multiple myeloma) cells, suggesting a universal effect across various hematologic cancer cell lines (Figure 1C).

**Figure 2:**
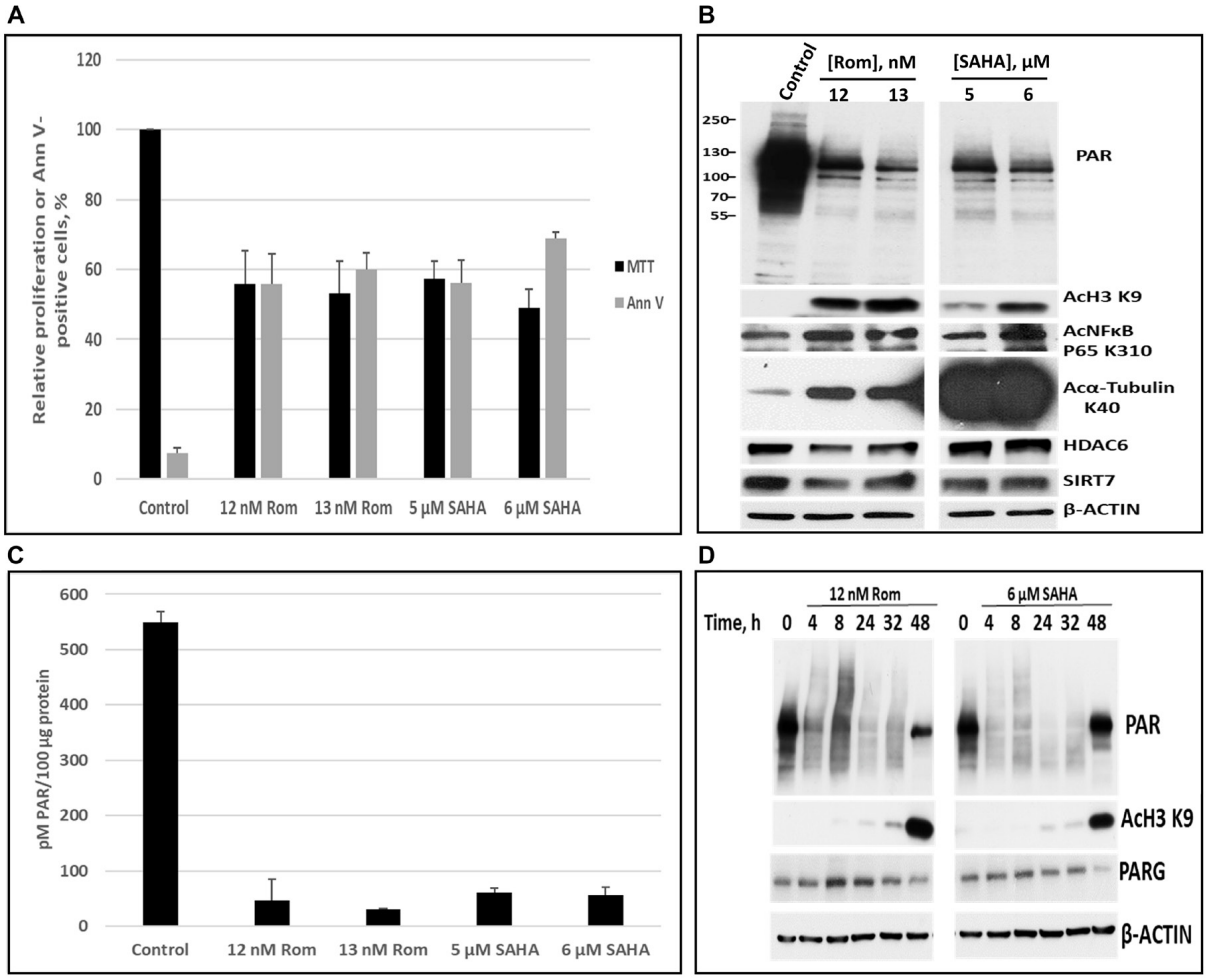
Comparison of the effects of romidepsin (Rom) and SAHA on protein poly(ADP-ribosyl)ation in the PEER T-cell leukemia cell line. Cells were exposed to the indicated drug concentrations for 48 h and analyzed by the MTT and Annexin V (AnnV) assays (**A**). The levels of poly(ADP-ribosyl)ation were determined by Western blotting (**B**) and ELISA (**C**). Protein levels and modifications were determined at various time points (**D**).

We then focused on PEER, the most sensitive cell line to Rom in terms of PARylation inhibition (Figure 1C). Again, MTT and Annexin V assays were used to determine the IC_50_ values of Rom and SAHA in the PEER cell line (Figure 2A). Using these concentrations, Rom and SAHA strongly inhibited PARylation (both by Western blot and ELISA), and caused acetylation of histone 3, NFκB and α-tubulin (Figure 2B, 2C). Rom substantially decreased the level of the histone deacetylases HDAC6 and SIRT7 whereas SAHA had minimal effects (Figure 2B). Figure 2D shows the kinetics of PARylation inhibition, histone acetylation, and level of poly(ADP-ribose) glycohydrolase (PARG), the major enzyme that removes poly(AD-Pribose), for both Rom and SAHA. Inhibition of PARylation, which occurred after 4-h drug exposure, preceded the acetylation of histone 3 at K9. The level of PARG enzyme decreased after 48-h exposure (Figure 2D).

### Inhibition of protein PARylation is caspase-independent

**Figure 3:**
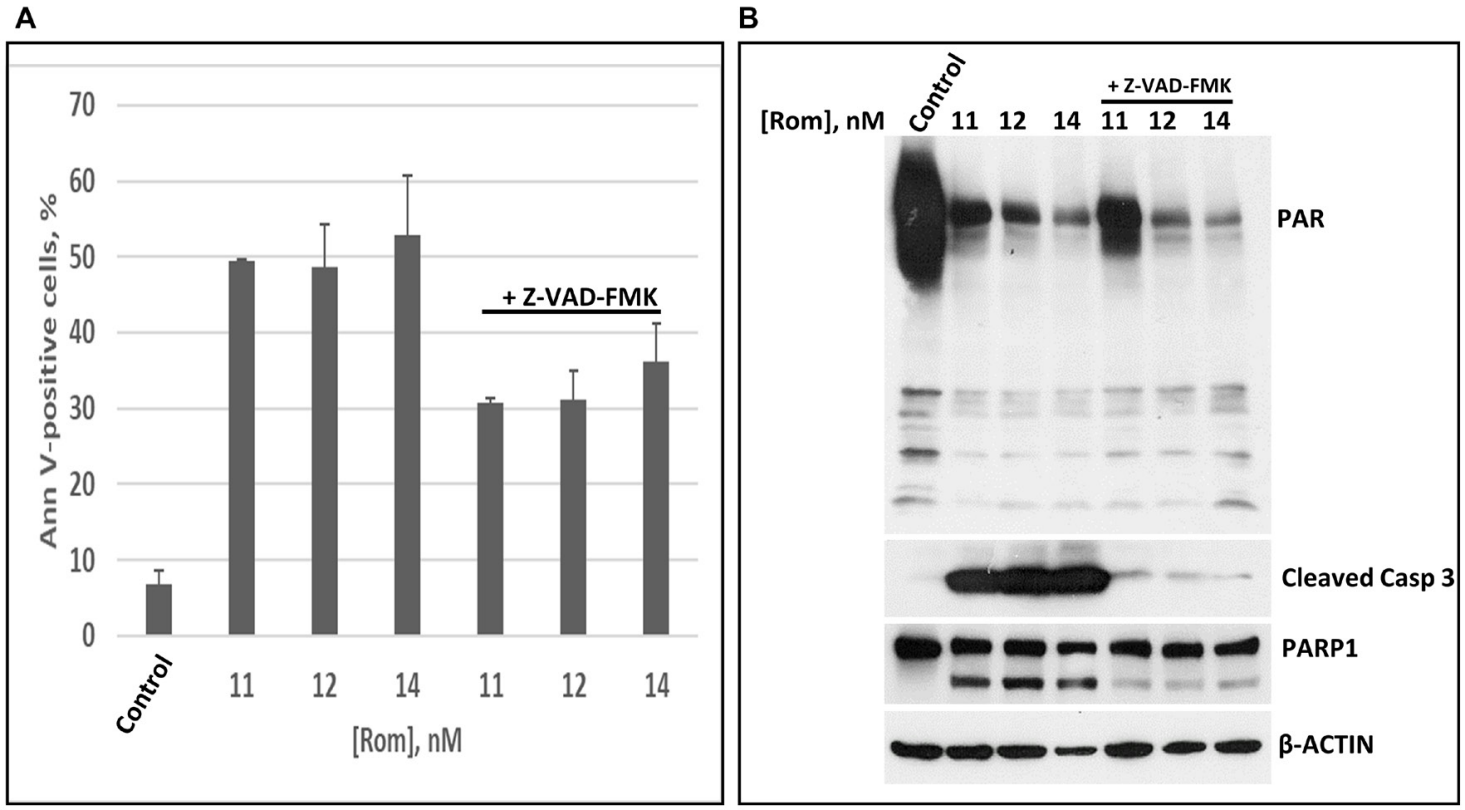
The inhibition of poly(ADP-ribosyl)ation is caspase independent. PEER cells were exposed to the indicated concentrations of romidepsin (Rom) with or without caspase inhibitor Z-VAD-FMK for 48 h prior to Annexin V (AnnV) (**A**) and Western blot (**B**) analyses. Abbreviation: Casp: Caspase.

HDACi activate Caspase 3 [21] and may lead to cleavage of PARP1 [22]. This cascade of events may contribute to the observed inhibition of PARylation. To determine if HDACi-mediated inhibition of PARylation is caspase-dependent, PEER cells were exposed to various concentrations of Rom in the absence or presence of 40 μM Z-VAD-FMK, a pan caspase inhibitor. Z-VAD-FMK decreased the proportion of Annexin V-positive cells due to Rom treatment (Figure 3A) and inhibited the cleavage of Caspase 3 (Figure 3B), but it did not relieve the Rom-mediated inhibition of PARylation (Figure 3B). These results indicate that the observed inhibition of PARylation due to Rom does not depend on Caspase 3 activity.

### HDACi inhibit protein PARylation in patient-derived cell samples

**Figure 4:**
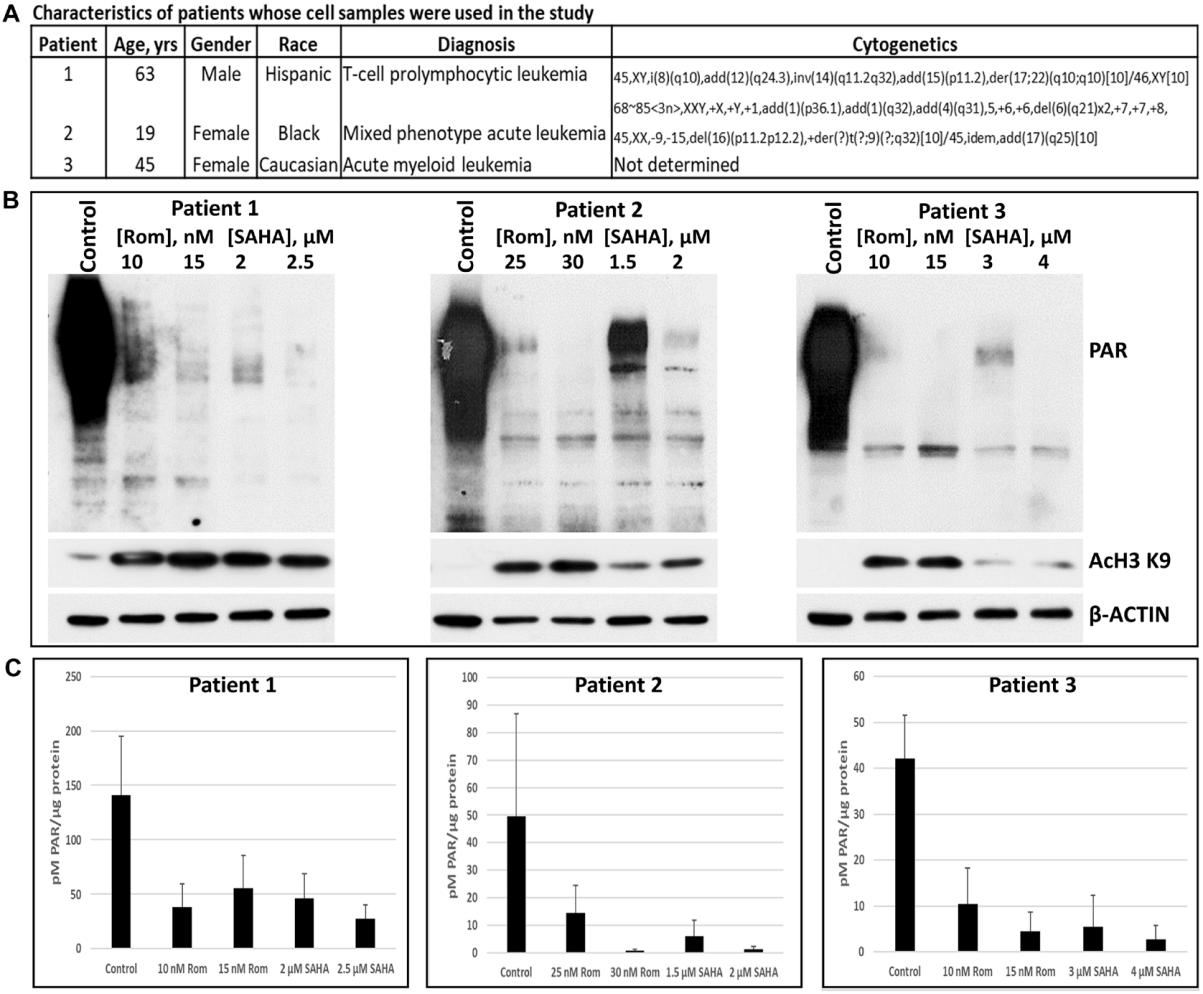
Effects of romidepsin (Rom) and SAHA on the levels of poly(ADP-ribosyl)ation in patient-derived cell samples. Mononuclear cells were isolated from peripheral blood of patients with hematologic malignancies (**A**) and exposed to the indicated drugs for 48 h prior to analysis by Western blotting (**B**) and ELISA (**C**).

To determine the potential clinical significance of our observations, we determined the effects of Rom and SAHA on cell samples derived from three leukemia patients (Figure 4A). The two HDACi induced acetylation of histone 3 at lysine 9 as expected (Figure 4B) and strongly inhibited global protein PARylation as evidenced both by Western blot analysis and ELISA (Figure 4B, 4C).

### The effect of HDACi on PARylation is mediated through PARP1

**Figure 5:**
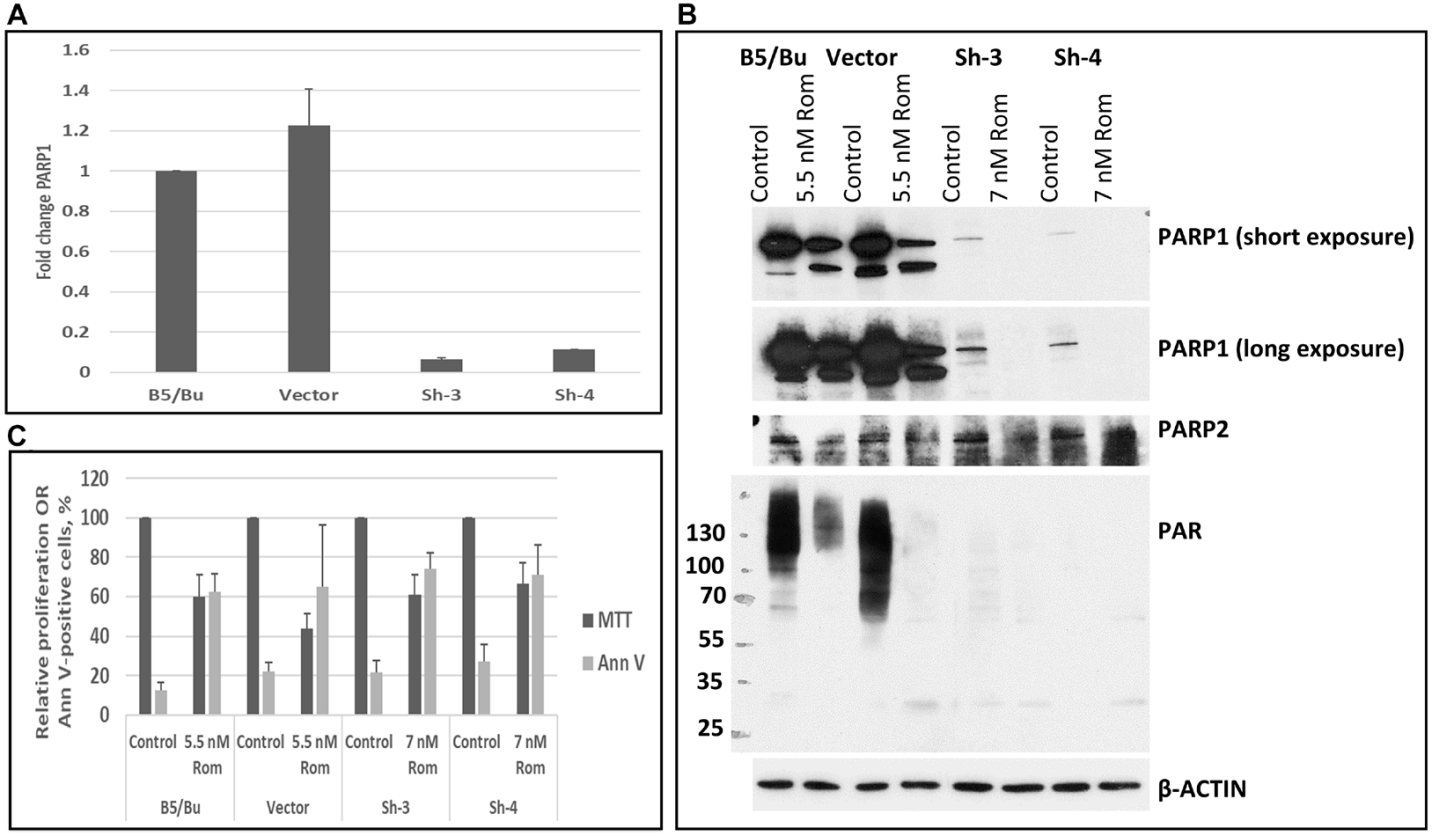
PARP1 is the major enzyme that catalyzes the poly(ADP-ribosyl)ation that is inhibited by romidepsin. KBM7/B5/Bu250^6^ (B5/Bu) CML cells were transduced with a lentivirus vector or lentivirus shRNA construct for PARP1, and stable clones (Sh-3 and Sh-4) were analyzed for expression of PARP1 by RT-PCR (**A**) and Western blotting (**B**). Cells were exposed to romidepsin (Rom) and analyzed by MTT and Annexin V (Ann V) assays (**C**). The numbers on the left side of panel B refer to the molecular weight markers (kDa).

To determine if the PARylation of proteins that is inhibited by HDACi is catalyzed by PARP1, the expression of the enzyme was knocked-down using shRNA lentivirus. A quantitative RT-PCR analysis showed decreased expression of PARP1 in two shRNA clones, Sh-3 and Sh-4 (Figure 5A). These two clones exhibited slight resistance to Rom; the IC_50_ values for the parental B5/Bu cells and vector-transduced cells were ~5–6 nM Rom and >7 nM Rom for the two shRNA clones (Figure 5B). Western blot analysis confirmed the down-regulation of PARP1 expression in the shRNA clones without a significant effect on PARP2 protein level (Figure 5C). PARylation in the PARP1-shRNA knockdown clones was remarkably low in the untreated control and abrogated by treatment with Rom, suggesting that PARP1 catalyzes the PARylation that is inhibited by HDACi.

### HDACi inhibit PARP1 at the transcription level

**Figure 6:**
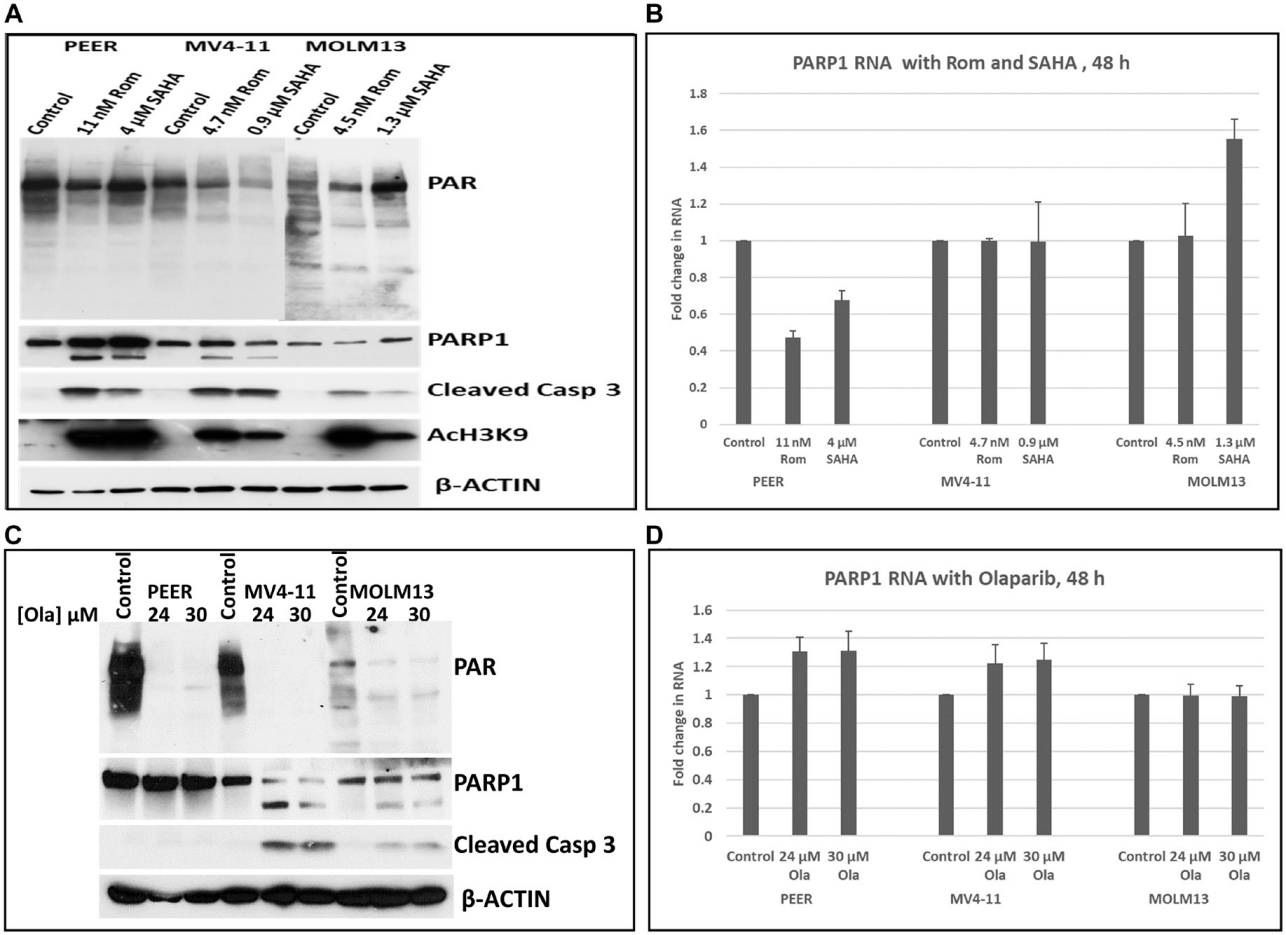
Effects of HDAC and PARP inhibitors on the transcription of the PARP1 gene. Cells were exposed to either romidepsin (Rom) or SAHA and the expression of PARP1 was analyzed by Western blotting (**A**) and RT-PCR (**B**). Cells were exposed to the indicated concentrations of olaparib (Ola) for 48 h and analyzed by Western blotting (**C**) and RT-PCR (**D**). Abbreviation: Casp: Caspase.

Epigenetic changes caused by HDACi are typically associated with alterations in gene expression [3, 4]. We, therefore, determined if HDACi affected the transcription of the *PARP1* gene. Cells from PEER, MV4-11 and MOLM13 cultures that were exposed to Rom and SAHA all showed inhibition of PARylation by Western blotting (Figure 6A). Partial cleavage of PARP1 in PEER and MV4-11 cells was observed after exposure to Rom and SAHA, but not in MOLM13 cells (Figure 6A). At equi-cytotoxic concentrations of each HDAC inhibitor in the three cell lines, quantitative RT-PCR analysis showed inhibition of *PARP1* gene transcription by both Rom and SAHA in PEER cells but not in MV4-11 and MOLM13 cells, suggesting that the effects of HDACi on *PARP1* gene transcription are cell line-dependent (Figure 6B). On the other hand, the PARPi Olaparib (Ola), which inhibits PARP1 primarily at the protein level by binding to and inhibiting the active catalytic site [23], strongly inhibited PARylation in these cell lines (Figure 6C) but did not affect the transcription of the *PARP1* gene (Figure 6D).

### HDACi down-regulate proteins involved in DNA repair

**Figure 7:**
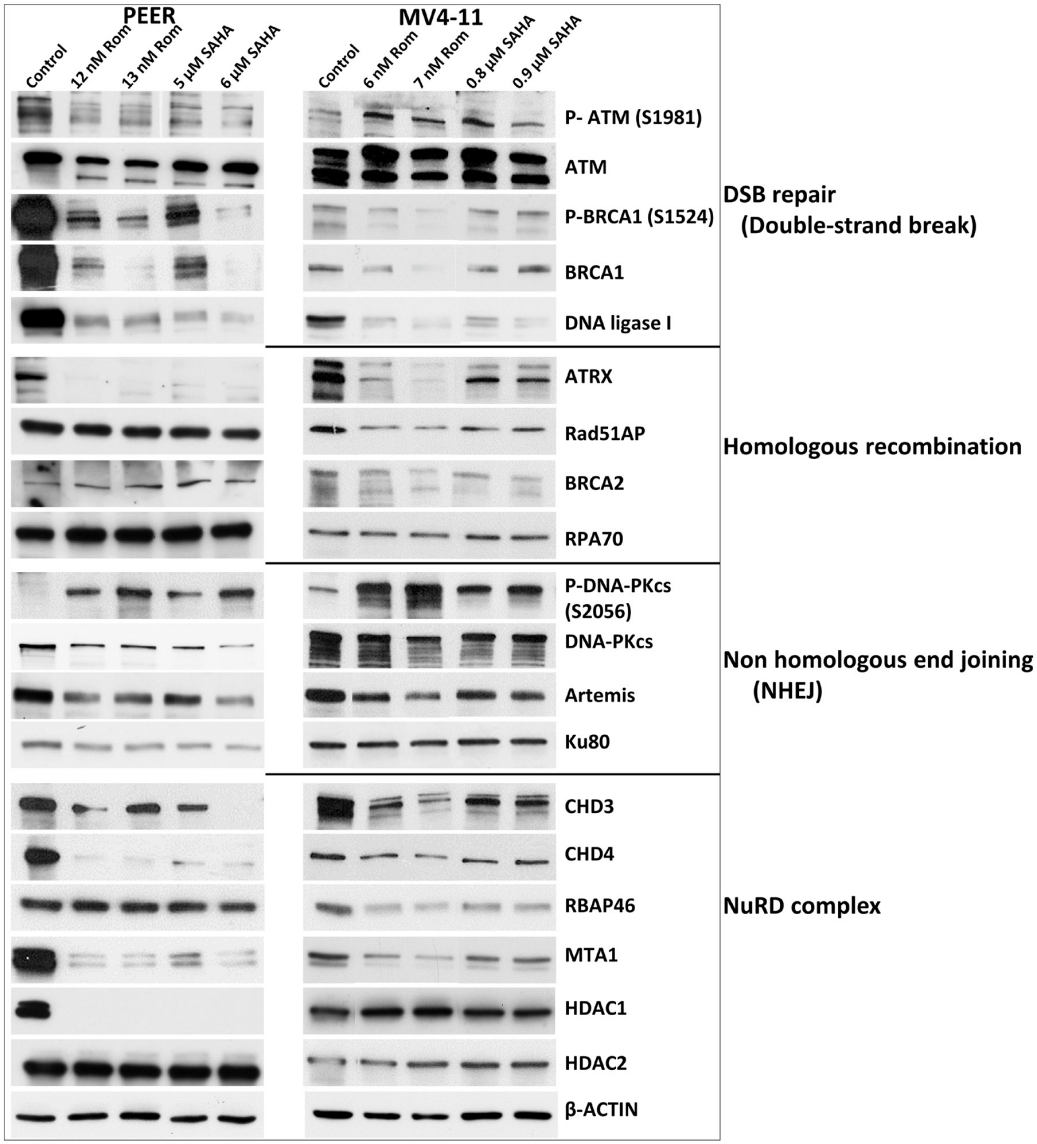
Effects of romidepsin (Rom) and SAHA on the levels and phosphorylation status of various proteins involved in DNA repair/DNA damage response in the PEER and MV4-11 cell lines. Cells were exposed to the indicated drug concentration for 48 h prior to analysis by Western blotting.

PARylation and acetylation are known to occur in some proteins involved in DNA repair [20, 24]. These post-translational modifications affect the stability of the proteins as previously shown for UHFR1 and BRCA1 [15, 19]. We, therefore, examined the effects of Rom and SAHA on their levels (total and phosphorylated) in the PEER and MV4-11 cell lines. While minimal changes were observed for ATM (which functions in DNA DSB repair), the level and phosphorylation of BRCA1 (which functions in homologous recombination (HR) repair) greatly decreased; the level of DNA ligase 1 (which functions in base excision repair and DNA replication) also significantly decreased (Figure 7).

ATRX is a chromatin remodeling protein involved in HR [25]. Both Rom and SAHA decreased the level of ATRX in PEER and MV4-11 cells with minimal effects on other HR proteins including Rad51AP, BRCA2 and RPA70 (Figure 7).

While the level of the non-homologous end joining (NHEJ) repair protein DNA-PKcs decreased in cells exposed to Rom and SAHA, its phosphorylation at serine 2056 remarkably increased; the levels of other NHEJ proteins - Artemis and Ku80 - slightly decreased (Figure 7).

The NuRD complex is involved in chromatin remodeling and deacetylation processes [26] and plays a key role in the cellular DNA damage response by regulating DNA damage signaling and repair events [27]. The levels of the CHD3, CHD4, and MTA1 subunits of NuRD decreased in both cell lines exposed to Rom and SAHA; the RBAP46 subunit decreased in MV4-11 but not in PEER cells and the HDAC1 subunit was obliterated in PEER cells but was unchanged in MV4-11 cells (Figure 7). HDAC2 was not affected by Rom or SAHA in either cell line.

### HDACi provide synergistic cytotoxicity with PARP inhibitors in hematologic cancer cells

**Figure 8:**
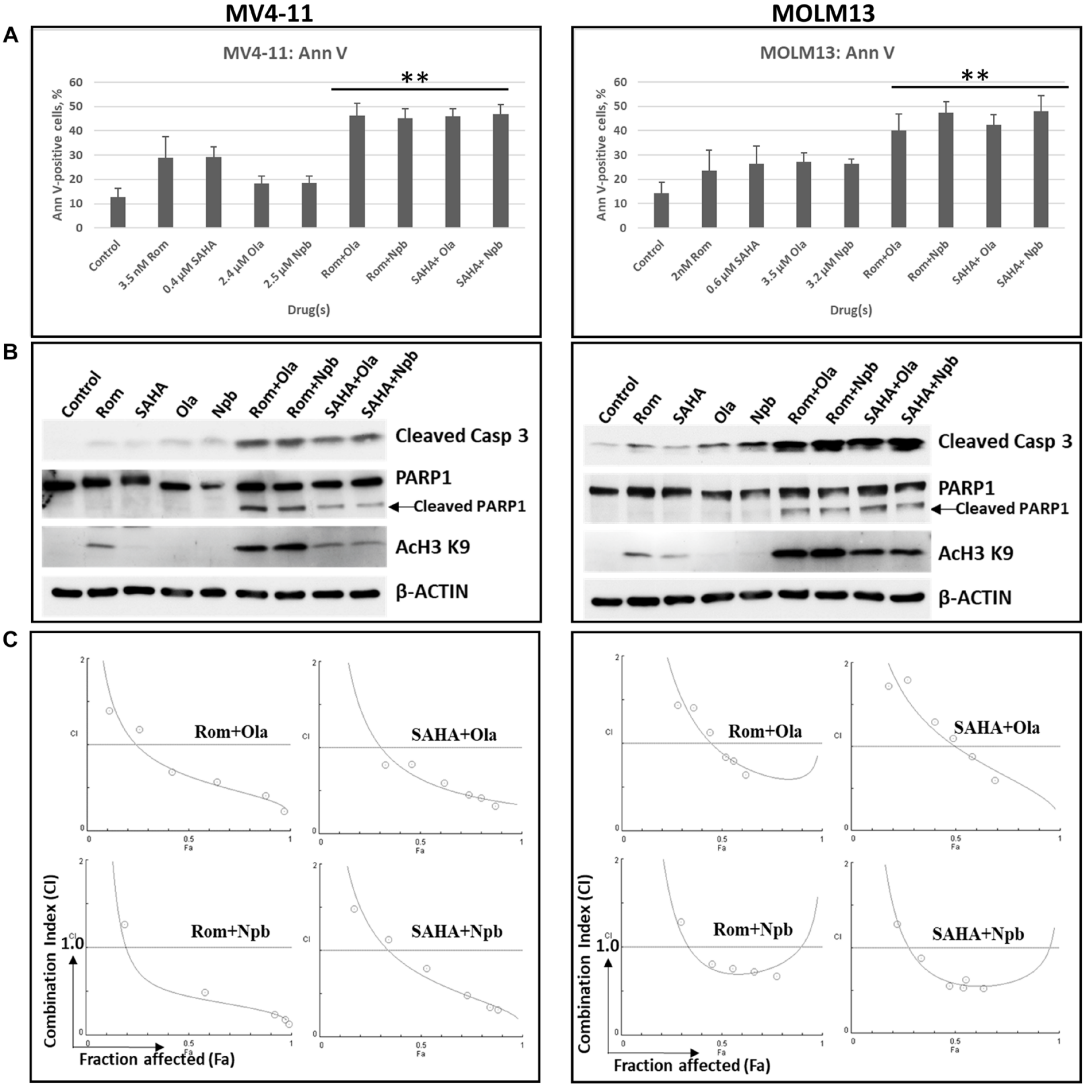
Synergistic cytotoxicity of HDAC and PARP inhibitors. Cells were exposed to drugs, individually or in combination, for 48 h and analyzed by (**A**) Annexin V (Ann V) assay or (**B**) Western blotting. (**C**) Cells were exposed to various concentrations of the indicated drugs at a constant ratio for 48 h and analyzed by MTT assay. Combination index (CI) values were calculated using the Chou and Talalay methodology. CI = 1 indicates additive effect; CI > 1 indicates antagonism; CI < 1 indicates synergism. ^**^Statistically significant (*P* ≤ 0.01) when compared with individual drug. Abbreviations: Rom: romidepsin; SAHA: vorinostat; Ola: olaparib; Npb: niraparib; Casp: Caspase.

The observed inhibition of PARylation mediated by HDACi highlights the strong potential for PARP and HDAC inhibitors to exhibit a synergistic activity in these hematologic cancer cell lines, as reported previously in cell lines derived from various types of human cancer [15–19, 28]. Cells were exposed to HDACi and PARPi, individually or in combination, and their effects on apoptosis (level of Ann V-positive cells and cleavage of Caspase 3) were determined. Exposure of MV4-11 and MOLM13 cells to individual HDACi (Rom, SAHA) or PARPi (Ola, niraparib (Npb)) slightly increased the proportion of Ann V-positive cells (Figure 8A) and cleavage of Caspase 3 (Figure 8B). These effects were significantly increased when the two classes of drugs were combined. Similar effects were observed for PARP1 cleavage and histone acetylation (Figure 8B). The calculated combination indexes for all drug combinations were less than 1.0, suggesting synergistic cytotoxicity (Figure 8C).

### Efficacy of combining HDAC and PARP inhibitors with chemotherapy drugs in lymphoma cells

**Figure 9:**
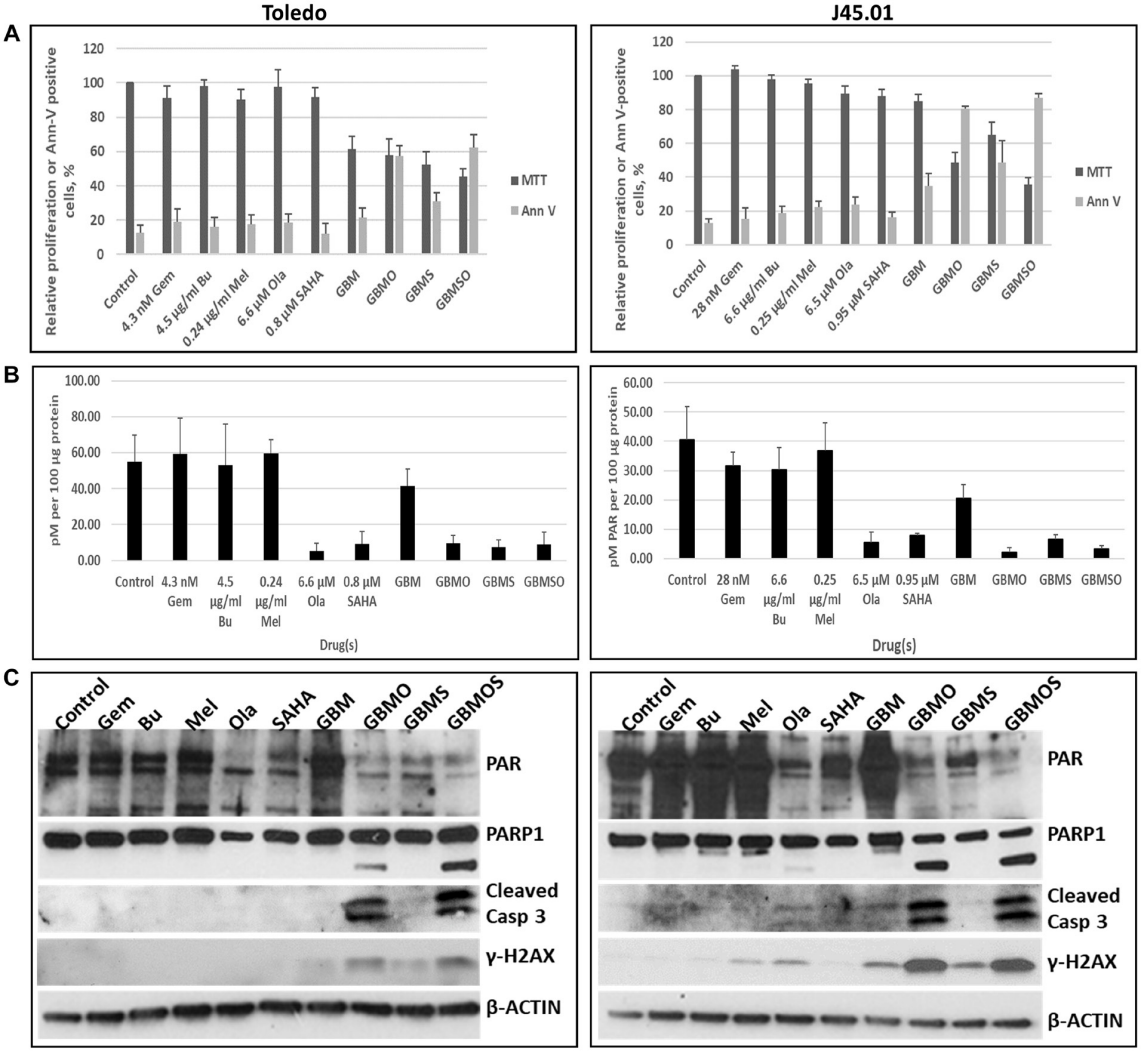
HDAC and PARP inhibitors enhance the cytotoxicity of nucleoside analog-alkylating agents in combination. Cells were exposed to drugs, individually or in combination, for 48 h and analyzed for proliferation (MTT assay) and activation of apoptosis (Annexin V (Ann V) assay) (**A**). The level of poly(ADP-ribosyl)ation was determined by ELISA (**B**). Western blotting was used to determine changes in the levels of PARylated proteins, PARP1 and Caspase 3 (Casp 3) cleavages, and γ-H2AX (**C**). Abbreviations: Gem: gemcitabine; Bu: busulfan; Mel: melphalan; Ola: olaparib; SAHA: vorinostat.

Both pre-clinical and clinical studies in our laboratory showed the efficacy of combined gemcitabine (Gem), busulfan (Bu) and melphalan (Mel) in inhibiting the proliferation of lymphoma cells [29, 30]. We sought to determine if addition of [HDACi + PARPi] to these chemotherapy drugs would enhance their cytotoxicity. Using drug concentrations close to their IC_15_ values, the combination of Gem, Bu, Mel, SAHA and Ola inhibited cell proliferation by ~55% and ~65% relative to the control in Toledo and J45.01 lymphoma cells, respectively, and Ann V-positivity increased to 60% and 85% (Figure 9A). As with the previously described cell lines, SAHA (HDACi) and Ola (PARPi) inhibited PARylation in these two lymphoma cell line models and their combination with Gem, Bu, and Mel did not affect this inhibition as shown by ELISA and Western blotting (Figure 9B, 9C). The 5-drug combination had more dramatic effects on the cleavages of PARP1 and Caspase 3 and on the phosphorylation of H2AX compared with the individual drugs or the [Gem+Bu+Mel] combination (Figure 9C). These results indicate a potential synergy in the efficacy of combining chemotherapy drugs with [HDACi+PARPi] for inhibition of lymphoma cell proliferation.

## DISCUSSION

The antitumor efficacy of HDACi involves several mechanisms that revolve around epigenetic regulation of gene expression by remodeling chromatin. The present study reports the HDACi-mediated inhibition of protein PARylation in hematologic cancer cell lines, which correlates with decreased levels and phosphorylation of major proteins involved in DNA repair. Such down-regulation of PARylation is seen across different hematologic cancer cell lines and also in patient-derived leukemic cell samples. This observation is consistent with the synergism of HDACi with agents that inhibit PARP1, the enzyme that catalyzes this PARylation.

Our study suggests that the observed HDACi-mediated inhibition of PARylation mainly involves PARP1 in our cell line models. Depletion of PARP1 using shRNA resulted in abrogation of protein PARylation, suggesting that PARP1 is the major enzyme that catalyzes PARylation, consistent with previous reports [31]. HDACi down-regulated the transcription of the *PARP1* gene in the PEER cell line but not in MV4-11 or MOLM13 cells (Figure 6B), suggesting that the effects on transcription are cell-context dependent. Exposure of cells to HDACi slightly decreased the level of PARP1 protein and caused its partial cleavage (Figures 1B, 5B, 6A), but such effects did not correlate with the significant decrease in PARylation observed in the same cells. This HDACi-mediated down-regulation of PARP1 is consistent with our previous clinical observations where isolated cell samples from patients treated with vorinostat/SAHA, Gem, Bu and Mel showed a marked decrease in the level of PARP1 protein [32].

PARylation of chromatin factors such as histones, topoisomerases and DNA repair proteins plays a critical role in the DNA damage response by modulating their localization, stability and activity [33, 34]. Our results show that the observed HDACi-mediated inhibition of PARylation correlates with changes in the status of proteins involved in DSB repair (HR, NHEJ) and the NuRD complex (Figure 7). HDACi are known to target DSB repair by regulating the acetylation status of key HR and NHEJ proteins [35].

There are other mechanisms through which HDACi may inhibit DNA repair. Exposure of HuT 78 cutaneous T-lymphocyte and LOX-IMVI melanoma cell lines to Rom resulted in chromatin hyperacetylation and caused accumulation of DNA-RNA hybrids (R-loops) which repressed transcription of genes involved in DNA repair and consequently provoked and amplified single-stranded DNA damage leading to cell death [36]. The selective HDAC1/2 inhibitor Entinostat inhibits HR repair by reducing BRCA1 expression and stalling replication fork progression, leading to irreparable DNA damage and ultimate cell death [37]. A recent study showed that HDACi sensitize HR-proficient human ovarian cancer cells to PARP inhibitors [38], and although the authors showed that Pano+Ola combination decreased cell viability and HR repair and enhanced DNA damage, the effects on PARylation were not determined.

At concentrations of HDACi that mediate partial inhibition of PARylation, it is intuitive that addition of PARP inhibitor would at least provide an additive cytotoxicity. Indeed, combination of HDAC and PARP inhibitors resulted in synergistic cytotoxicity in the hematologic cancer cell lines studied here (Figure 8). It is possible that HDACi increase the acetylation of proteins which consequently blocks their ADP-ribosylation, as has been shown for histone H3 [20]. Moreover, combination of HDACi and PARPi have been shown to cause PARP1 trapping to chromatin and could have resulted in the inhibition of its enzymatic activity [39]. The observed HDACi-mediated down-regulation of key DNA repair proteins (Figure 7) combined with the high levels of oxidative and replication stress characteristic of many human cancers could have made these cancer cells not only very sensitive to HDACi via DNA repair inhibition but also highly dependent on PARP and therefore extremely sensitive to combined HDACi/PARPi treatment.

The synergistic effect of HDACi and PARPi and the implied magnified inhibition of DNA repair also provides a platform for combining these inhibitors with chemotherapeutic agents as previously shown for triple negative breast cancers when such inhibitors were combined with cisplatin [15]. Our results show that combination of [Ola+SAHA] with [Gem+Bu+Mel] efficiently inhibited cell proliferation and activated apoptosis in lymphoma cell line models (Figure 9). The cytotoxicity of the [HDACi+PARPi+Gem+Bu+Mel] combination might be mediated in part at the level of the initial DNA damage invoked by the nucleoside analog and two DNA alkylating agents, as we previously showed [29]. Repair of the damaged DNA is known to require the initial binding of PARP1 at the damage sites [40], which poly(ADP-ribosyl)ates itself, histones and certain chromatin-associated proteins; the PARylated complex facilitates chromatin remodeling and provides a scaffold for recruitment of the DNA repair machinery [41]. This process could be abrogated by the combined effects of HDACi and PARPi through inhibition of PARylation. These results are consistent with the clinical efficacy of [SAHA/vorinostat+Gem+Bu+Mel] with autologous stem cell transplantation in patients with refractory lymphomas [32], which may also be partly due to SAHA-mediated inhibition of PARylation and down-regulation of MRP1 protein, a transporter for Bu and Mel [10].

A possible clinical efficacy of [HDACi+PARPi+Gem+Bu+Mel] combination was demonstrated by our preliminary results, which showed an inhibition of PARylation in mononuclear cells obtained from lymphoma patients enrolled in our ongoing clinical trial (data not shown). A detailed report on this study will be presented in a separate manuscript.

In conclusion, our results provide a molecular explanation for the HDACi-mediated inhibition of DNA repair in hematologic cancer cells and support the combinatorial application of HDACi, PARPi and chemotherapeutic agents for the treatment of hematologic malignancies.

## MATERIALS AND METHODS

### Cell lines and drugs

The MV4-11 and MOLM13 AML cell lines were kindly provided by Dr. Michael Andreeff’s laboratory (University of Texas MD Anderson Cancer Center, Houston, TX, USA). PEER (from Dr. Guillermo Garcia-Manero’s laboratory, University of Texas MD Anderson Cancer Center) is an established cell line originally isolated from patients with T-cell acute lymphoblastic leukemia. The busulfan-resistant KBM7/B5/Bu250^6^ chronic myeloid leukemia (CML) cell line was established in our laboratory as described previously [42]. The two lymphoma cell line models J45.01 and Toledo, and the RPMI8226 multiple myeloma cell line, were purchased from the American Type Culture Collection (Manassas, VA, USA). Cells were grown in Roswell Park Memorial Institute medium 1640 (Mediatech, Manassas, VA, USA) supplemented with 10% heat-inactivated fetal bovine serum (FBS: Gemini Bio-products, West Sacramento, CA, USA) and 100 IU/mL penicillin and 100 μg/mL streptomycin (Mediatech) at 37°C in a humidified atmosphere of 5% CO_2_ in air.

The following drugs were purchased from Selleck Chemicals (Houston, TX, USA): suberoylanilide hydroxamic acid (SAHA or Vorinostat), panobinostat (Pano), romidepsin (Rom), trichostatin A (TSA), olaparib (Ola), niraparib (Npb), gemcitabine (Gem) and Z-VAD-FMK. The stock solutions of all drugs including busulfan and melphalan (Sigma-Aldrich, St. Louis, MO, USA) were prepared in dimethyl sulfoxide (DMSO). The final concentration of DMSO in all experiments did not exceed 0.08% by volume, a level that does not induce differentiation of these cell lines.

### Patient samples

Mononuclear cells were purified from patient-derived cell samples using lymphocyte separation medium (Mediatech) and incubated in suspension in Roswell Park Memorial Institute 1640 medium as described above. The samples were obtained after obtaining written informed consent, and all studies using these patient samples were performed under a protocol approved by the Institutional Review Board of the University of Texas MD Anderson Cancer Center, in accordance with the Declaration of Helsinki.

### Cell proliferation and cell death assays

Cell proliferation was determined using the 3-(4,5-dimethylthiazol-2-yl)-2,5-diphenyl tetrazolium bromide (MTT) assay. The inhibition of cell proliferation after 48-h drug exposure was determined relative to the control cells exposed to solvent alone. The IC_50_ value (the concentration of drug that inhibited 50% proliferation) was calculated using the CalcuSyn software (Biosoft, Ferguson, MO, USA). Cell death was determined by flow cytometric measurements of phosphatidylserine externalization with Annexin-V-FLUOS (Roche Diagnostics, Indianapolis, IN, USA) and 7-aminoactinomycin D (BD Biosciences, San Jose, CA, USA) using a Muse Cell Analyzer (MilliporeSigma, St. Louis, MO, USA).

Drug combination effects were estimated based on the combination index (CI) values calculated using the CalcuSyn software (Biosoft). This program was developed based on the median-effect method: CI < 1 indicates synergy, CI ≈ 1 is additive, and CI > 1 suggests antagonism.

### Western blot analysis

Cells were exposed continuously to drug(s) for 48 h, harvested and washed with cold phosphate-buffered saline. Cells were lysed with lysis buffer (Cell Signaling Technology, Danvers, MA, USA). Total protein concentrations in the cell lysates were determined using a BCA Protein Assay kit (Thermo Fisher Scientific, Rockford, IL, USA). Western blot analysis was done by separating protein extracts on polyacrylamide-SDS gels and blotting onto nitrocellulose membranes (Bio-Rad, Hercules, CA, USA). Immunoblot analyses by chemiluminescence were done using the Immobilon Western Chemiluminescent HRP Substrate (MilliporeSigma). The sources of the antibodies and their optimum dilutions are available upon request. The β-actin protein was used as an internal control.

### Determination of the level of poly(ADP-ribosyl)ation

The levels of total PARylated proteins were determined by Western blot analysis (as described above) and enzyme-linked immunosorbent assay (ELISA) using the poly(ADP-Ribose) ELISA kit from Cell Biolabs, Inc. (San Diego, CA, USA). The monoclonal anti-PAR antibody used for Western blotting was obtained from R&D Systems, Inc. (Minneapolis, MN, USA). The antibody is specific for PAR polymers 2 to 50 units long, but does not recognize structurally related RNA, DNA, ADP-ribose monomers, NAD, or other nucleic acid monomers.

### shRNA lentiviral particle transduction

Control vector (sc-108080) and PARP1 (sc-29437-v) shRNA lentiviral particles were purchased from Santa Cruz Biotechnology, Inc. (Dallas, TX, USA). In a 96-well plate, KBM7/B5/Bu250^6^ cells (5 × 10^6^ cells in 50 μl medium) were mixed with 50 μl viral suspension and Polybrene at a final concentration of 5 μg/ml. The plate was centrifuged at 2000 × g for 90 min at 30°C. Complete medium (150 μl) was added per well and incubated at 37°C, 5% CO_2_ for 3 h. The cells were then washed with the medium twice and resuspended in 3 ml medium in a 6-well plate. After 24-h incubation, cells were centrifuged and resuspended in 6 ml medium and transferred to a T25 flask for another 24-h incubation. Selection was started with 4 μg/ml puromycin, which was gradually increased to 5 and 6 μg/ml at 3-day intervals. Stable clones were purified by serial dilution in the presence of 6 μg/ml puromycin and analyzed for decreased expression of PARP1 by Western blotting.

### Quantitative real-time PCR

Real-time PCR was used to determine the level of expression of PARP1. Total RNA was extracted from cells exposed to the indicated drug(s) for 48 h using the RNeasy Mini Kit (QIAGEN, Valencia, CA, USA) and used for complementary DNA synthesis using the High Capacity Reverse Transcription kit (Applied Biosystems, Foster City, CA, USA). Quantitative real-time PCR was done using TaqMan^™^ Gene Expression Assays for GAPDH (Hs02786624_g1) and PARP1 (Hs00911377_g1) and the TaqMan^™^ Fast Advanced Master Mix from Applied Biosystems. The amplification method included initial heating at 95°C for 2 min, followed by 40 cycles of 95°C for 3 sec and annealing temperature of 60°C for 32 sec using the 7500 Real Time PCR System (Applied Biosystems). The quantification of gene expression was carried out by comparative CT methodology using the GAPDH gene as an internal control. Fold-change in the level of PARP1 expression was calculated using the 2^−ΔΔCT^ method, where ΔΔC_T_ = (C_T,PARP1_− C_T,GAPDH_)_drug X_ – (C_T,PARP1_ − C_T,GAPDH_)_Control_.

### Statistical analysis

Results are presented as the mean ± s.d. of at least three independent experiments and statistical analysis was performed using a Student’s paired *t*-test with a two-tailed distribution.

## Abbreviations

AML: acute myeloid leukemia
Ann V: annexin V
Bu: busulfan
CI: combination index
CML: chronic myeloid leukemia
CT: threshold cycle
DMSO: dimethyl sulfoxide
DSB: double-strand break
γ-H2AX: phosphorylated histone 2AX
Gem: gemcitabine
h: hour/hours
HDACi: histone deacetylase inhibitor
HR: homologous recombination
Mel: melphalan
min: minutes
MTT: 3-(4,5-dimethylthiazol-2-yl)-2,5-diphenyl tetrazolium bromide
NHEJ: non-homologous end joining
Npb: niraparib
Ola: olaparib
Pano: panobinostat
PARylation: poly(ADP-ribosyl)ation
PARPi: poly(ADP-ribose) polymerase inhibitor
Rom: romidepsin
SAHA: suberoylanilide hydroxamic acid/vorinostat
TSA: trichostatin A

## References

[1] Kuo MH , Allis CD . Roles of histone acetyltransferases and deacetylases in gene regulation. Bioessays. 1998; 20:615–26. 10.1002/(SICI)1521-1878(199808)20:8<615::AID-BIES4>3.0.CO;2-H. 9780836

[2] Turner BM . Histone acetylation and an epigenetic code. Bioessays. 2000; 22:836–45. 10.1002/1521-1878(200009)22:9<836::AID-BIES9>3.0.CO;2-X. 10944586

[3] Glozak MA , Seto E . Histone deacetylases and cancer. Oncogene. 2007; 26:5420–32. 10.1038/sj.onc.1210610. 17694083

[4] Cohen I , Poręba E , Kamieniarz K , Schneider R . Histone modifiers in cancer: friends or foes? Genes Cancer. 2011; 2:631–47. 10.1177/1947601911417176. 21941619 PMC3174261

[5] Yoon S , Eom GH . HDAC and HDAC Inhibitor: From Cancer to Cardiovascular Diseases. Chonnam Med J. 2016; 52:1–11. 10.4068/cmj.2016.52.1.1. 26865995 PMC4742605

[6] Kim HJ , Bae SC . Histone deacetylase inhibitors: molecular mechanisms of action and clinical trials as anti-cancer drugs. Am J Transl Res. 2011; 3:166–79. 21416059 PMC3056563

[7] Montalban-Bravo G , Garcia-Manero G . Novel drugs for older patients with acute myeloid leukemia. Leukemia. 2015; 29:760–69. 10.1038/leu.2014.244. 25142817

[8] Nieto Y , Valdez BC , Thall PF , Jones RB , Wei W , Myers A , Hosing C , Ahmed S , Popat U , Shpall EJ , Qazilbash M , Gulbis A , Anderlini P , et al. Double epigenetic modulation of high-dose chemotherapy with azacitidine and vorinostat for patients with refractory or poor-risk relapsed lymphoma. Cancer. 2016; 122:2680–88. 10.1002/cncr.30100. 27203405 PMC4992444

[9] San José-Enériz E , Gimenez-Camino N , Agirre X , Prosper F . HDAC Inhibitors in Acute Myeloid Leukemia. Cancers (Basel). 2019; 11:1794. 10.3390/cancers11111794. 31739588 PMC6896008

[10] Valdez BC , Li Y , Murray D , Brammer JE , Liu Y , Hosing C , Nieto Y , Champlin RE , Andersson BS . Differential effects of histone deacetylase inhibitors on cellular drug transporters and their implications for using epigenetic modifiers in combination chemotherapy. Oncotarget. 2016; 7:63829–38. 10.18632/oncotarget.11561. 27564097 PMC5325407

[11] Bachy E , Camus V , Thieblemont C , Sibon D , Casasnovas RO , Ysebaert L , Damaj G , Guidez S , Pica GM , Kim WS , Lim ST , André M , García-Sancho AM , et al. Romidepsin Plus CHOP Versus CHOP in Patients With Previously Untreated Peripheral T-Cell Lymphoma: Results of the Ro-CHOP Phase III Study (Conducted by LYSA). J Clin Oncol. 2022; 40:242–51. 10.1200/JCO.21.01815. 34843406

[12] Bakkenist CJ , Kastan MB . DNA damage activates ATM through intermolecular autophosphorylation and dimer dissociation. Nature. 2003; 421:499–506. 10.1038/nature01368. 12556884

[13] Tang J , Cho NW , Cui G , Manion EM , Shanbhag NM , Botuyan MV , Mer G , Greenberg RA . Acetylation limits 53BP1 association with damaged chromatin to promote homologous recombination. Nat Struct Mol Biol. 2013; 20:317–25. 10.1038/nsmb.2499. 23377543 PMC3594358

[14] Gkotzamanidou M , Terpou E , Kentepozidis N , Terpos E . Targeting the Interplay between HDACs and DNA Damage Repair for Myeloma Therapy. Int J Mol Sci. 2021; 22:10406. 10.3390/ijms221910406. 34638744 PMC8508842

[15] Ha K , Fiskus W , Choi DS , Bhaskara S , Cerchietti L , Devaraj SG , Shah B , Sharma S , Chang JC , Melnick AM , Hiebert S , Bhalla KN . Histone deacetylase inhibitor treatment induces ‘BRCAness’ and synergistic lethality with PARP inhibitor and cisplatin against human triple negative breast cancer cells. Oncotarget. 2014; 5:5637–50. 10.18632/oncotarget.2154. 25026298 PMC4170637

[16] Baldan F , Mio C , Allegri L , Puppin C , Russo D , Filetti S , Damante G . Synergy between HDAC and PARP Inhibitors on Proliferation of a Human Anaplastic Thyroid Cancer-Derived Cell Line. Int J Endocrinol. 2015; 2015:978371. 10.1155/2015/978371. 25705225 PMC4326215

[17] Min A , Im SA , Kim DK , Song SH , Kim HJ , Lee KH , Kim TY , Han SW , Oh DY , Kim TY , O’Connor MJ , Bang YJ . Histone deacetylase inhibitor, suberoylanilide hydroxamic acid (SAHA), enhances anti-tumor effects of the poly (ADP-ribose) polymerase (PARP) inhibitor olaparib in triple-negative breast cancer cells. Breast Cancer Res. 2015; 17:33. 10.1186/s13058-015-0534-y. 25888415 PMC4425881

[18] Rasmussen RD , Gajjar MK , Jensen KE , Hamerlik P . Enhanced efficacy of combined HDAC and PARP targeting in glioblastoma. Mol Oncol. 2016; 10:751–63. 10.1016/j.molonc.2015.12.014. 26794465 PMC5423160

[19] Yin L , Liu Y , Peng Y , Peng Y , Yu X , Gao Y , Yuan B , Zhu Q , Cao T , He L , Gong Z , Sun L , Fan X , Li X . Correction to: PARP inhibitor veliparib and HDAC inhibitor SAHA synergistically co-target the UHRF1/. BRCA1 DNA damage repair complex in prostate cancer cells. J Exp Clin Cancer Res. 2022; 41:72. 10.1186/s13046-022-02290-9. 35193658 PMC8862322

[20] Liszczak G , Diehl KL , Dann GP , Muir TW . Acetylation blocks DNA damage-induced chromatin ADP-ribosylation. Nat Chem Biol. 2018; 14:837–40. 10.1038/s41589-018-0097-1. 30013063 PMC6505472

[21] Marks PA , Jiang X . Histone deacetylase inhibitors in programmed cell death and cancer therapy. Cell Cycle. 2005; 4:549–51. 10.4161/cc.4.4.1564. 15738652

[22] Rosen A , Casciola-Rosen L . Macromolecular substrates for the ICE-like proteases during apoptosis. J Cell Biochem. 1997; 64:50–54. 10.1002/(sici)1097-4644(199701)64:1<50::aid-jcb8>3.0.co;2-z. 9015754

[23] Goulooze SC , Cohen AF , Rissmann R . Olaparib. Br J Clin Pharmacol. 2016; 81:171–73. 10.1111/bcp.12761. 26344419 PMC4693566

[24] Curtin NJ , Szabo C . Poly(ADP-ribose) polymerase inhibition: past, present and future. Nat Rev Drug Discov. 2020; 19:711–36. 10.1038/s41573-020-0076-6. 32884152

[25] Elbakry A , Löbrich M . Homologous Recombination Subpathways: A Tangle to Resolve. Front Genet. 2021; 12:723847. 10.3389/fgene.2021.723847. 34408777 PMC8365153

[26] Gursoy-Yuzugullu O , House N , Price BD . Patching Broken DNA: Nucleosome Dynamics and the Repair of DNA Breaks. J Mol Biol. 2016; 428:1846–60. 10.1016/j.jmb.2015.11.021. 26625977 PMC4860187

[27] Smeenk G , Wiegant WW , Vrolijk H , Solari AP , Pastink A , van Attikum H . The NuRD chromatin-remodeling complex regulates signaling and repair of DNA damage. J Cell Biol. 2010; 190:741–49. 10.1083/jcb.201001048. 20805320 PMC2935570

[28] Kruglov O , Wu X , Hwang ST , Akilov OE . The synergistic proapoptotic effect of PARP-1 and HDAC inhibition in cutaneous T-cell lymphoma is mediated via Blimp-1. Blood Adv. 2020; 4:4788–97. 10.1182/bloodadvances.2020002049. 33017467 PMC7556155

[29] Valdez BC , Nieto Y , Murray D , Li Y , Wang G , Champlin RE , Andersson BS . Epigenetic modifiers enhance the synergistic cytotoxicity of combined nucleoside analog-DNA alkylating agents in lymphoma cell lines. Exp Hematol. 2012; 40:800–10. 10.1016/j.exphem.2012.06.001. 22687754 PMC3447105

[30] Nieto Y , Thall PF , Ma J , Valdez BC , Ahmed S , Anderlini P , Popat U , Jones RB , Shpall EJ , Hosing C , Qazilbash M , Kebriaei P , Alousi A , et al. Phase II Trial of High-Dose Gemcitabine/Busulfan/Melphalan with Autologous Stem Cell Transplantation for Primary Refractory or Poor-Risk Relapsed Hodgkin Lymphoma. Biol Blood Marrow Transplant. 2018; 24:1602–09. 10.1016/j.bbmt.2018.02.020. 29501779 PMC8212703

[31] Gibson BA , Kraus WL . New insights into the molecular and cellular functions of poly(ADP-ribose) and PARPs. Nat Rev Mol Cell Biol. 2012; 13:411–24. 10.1038/nrm3376. 22713970

[32] Nieto Y , Valdez BC , Thall PF , Ahmed S , Jones RB , Hosing C , Popat U , Shpall EJ , Qazilbash M , Gulbis A , Anderlini P , Alousi A , Shah N , et al. Vorinostat Combined with High-Dose Gemcitabine, Busulfan, and Melphalan with Autologous Stem Cell Transplantation in Patients with Refractory Lymphomas. Biol Blood Marrow Transplant. 2015; 21:1914–20. 10.1016/j.bbmt.2015.06.003. 26071868 PMC4781754

[33] Caldecott KW . Protein ADP-ribosylation and the cellular response to DNA strand breaks. DNA Repair (Amst). 2014; 19:108–13. 10.1016/j.dnarep.2014.03.021. 24755000

[34] Tallis M , Morra R , Barkauskaite E , Ahel I . Poly(ADP-ribosyl)ation in regulation of chromatin structure and the DNA damage response. Chromosoma. 2014; 123:79–90. 10.1007/s00412-013-0442-9. 24162931

[35] Wang X , Zhao J . Targeted Cancer Therapy Based on Acetylation and Deacetylation of Key Proteins Involved in Double-Strand Break Repair. Cancer Manag Res. 2022; 14:259–71. 10.2147/CMAR.S346052. 35115826 PMC8800007

[36] Safari M , Litman T , Robey RW , Aguilera A , Chakraborty AR , Reinhold WC , Basseville A , Petrukhin L , Scotto L , O’Connor OA , Pommier Y , Fojo AT , Bates SE . R-Loop-Mediated ssDNA Breaks Accumulate Following Short-Term Exposure to the HDAC Inhibitor Romidepsin. Mol Cancer Res. 2021; 19:1361–74. 10.1158/1541-7786.MCR-20-0833. 34050002 PMC8974437

[37] Gupta VG , Hirst J , Petersen S , Roby KF , Kusch M , Zhou H , Clive ML , Jewell A , Pathak HB , Godwin AK , Wilson AJ , Crispens MA , Cybulla E , et al. Entinostat, a selective HDAC1/2 inhibitor, potentiates the effects of olaparib in homologous recombination proficient ovarian cancer. Gynecol Oncol. 2021; 162:163–72. 10.1016/j.ygyno.2021.04.015. 33867143 PMC8647995

[38] Wilson AJ , Gupta VG , Liu Q , Yull F , Crispens MA , Khabele D . Panobinostat enhances olaparib efficacy by modifying expression of homologous recombination repair and immune transcripts in ovarian cancer. Neoplasia. 2022; 24:63–75. 10.1016/j.neo.2021.12.002. 34933276 PMC8702851

[39] Robert C , Nagaria PK , Pawar N , Adewuyi A , Gojo I , Meyers DJ , Cole PA , Rassool FV . Histone deacetylase inhibitors decrease NHEJ both by acetylation of repair factors and trapping of PARP1 at DNA double-strand breaks in chromatin. Leuk Res. 2016; 45:14–23. 10.1016/j.leukres.2016.03.007. 27064363 PMC5007632

[40] Schützenhofer K , Rack JGM , Ahel I . The Making and Breaking of Serine-ADP-Ribosylation in the DNA Damage Response. Front Cell Dev Biol. 2021; 9:745922. 10.3389/fcell.2021.745922. 34869334 PMC8634249

[41] De Vos M , Schreiber V , Dantzer F . The diverse roles and clinical relevance of PARPs in DNA damage repair: current state of the art. Biochem Pharmacol. 2012; 84:137–46. 10.1016/j.bcp.2012.03.018. 22469522

[42] Valdez BC , Murray D , Ramdas L , de Lima M , Jones R , Kornblau S , Betancourt D , Li Y , Champlin RE , Andersson BS . Altered gene expression in busulfan-resistant human myeloid leukemia. Leuk Res. 2008; 32:1684–97. 10.1016/j.leukres.2008.01.016. 18339423 PMC2633244

